# Biochemical characterization of a novel oxidatively stable, halotolerant, and high‐alkaline subtilisin from *Alkalihalobacillus okhensis* Kh10‐101^T^

**DOI:** 10.1002/2211-5463.13457

**Published:** 2022-07-06

**Authors:** Fabian Falkenberg, Jade Rahba, David Fischer, Michael Bott, Johannes Bongaerts, Petra Siegert

**Affiliations:** ^1^ Institute of Nano‐ and Biotechnologies Aachen University of Applied Sciences Jülich Germany; ^2^ Institute of Bio‐ and Geosciences IBG‐1: Biotechnology, Forschungszentrum Jülich Germany

**Keywords:** *Alkalihalobacillus okhensis*, detergent protease, halotolerant protease, high‐alkaline subtilisin, oxidative stable protease

## Abstract

Halophilic and halotolerant microorganisms represent a promising source of salt‐tolerant enzymes suitable for various biotechnological applications where high salt concentrations would otherwise limit enzymatic activity. Considering the current growing enzyme market and the need for more efficient and new biocatalysts, the present study aimed at the characterization of a high‐alkaline subtilisin from *Alkalihalobacillus okhensis* Kh10‐101^T^. The protease gene was cloned and expressed in *Bacillus subtilis* DB104. The recombinant protease SPAO with 269 amino acids belongs to the subfamily of high‐alkaline subtilisins. The biochemical characteristics of purified SPAO were analyzed in comparison with subtilisin Carlsberg, Savinase, and BPN'. SPAO, a monomer with a molecular mass of 27.1 kDa, was active over a wide range of pH 6.0–12.0 and temperature 20–80 °C, optimally at pH 9.0–9.5 and 55 °C. The protease is highly oxidatively stable to hydrogen peroxide and retained 58% of residual activity when incubated at 10 °C with 5% (v/v) H_2_O_2_ for 1 h while stimulated at 1% (v/v) H_2_O_2_. Furthermore, SPAO was very stable and active at NaCl concentrations up to 5.0 m. This study demonstrates the potential of SPAO for biotechnological applications in the future.

## Graphical abstract

A subtilisin‐type protease from *Alkalihalobacillus okhensis* Kh10‐101^T^ was cloned and expressed in *Bacillus subtilis* DB104. The comprehensive biochemical characterization revealed a resilient protease with high stability against hydrogen peroxide 5% (v/v) and activity at NaCl concentrations up to 5.0 m. The activity over a wide range of pH and temperature demonstrates the potential for biotechnological applications.

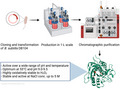

**Abbreviations**
aa: amino acid
*aprE*
: extracellular alkaline protease gene
CHCA: α‐Cyano‐4‐hydroxycinnamic acid
EDTA: ethylenediaminetetraacetic acid
IEF: isoelectric focussing
LB: lysogeny broth
LM: length marker
MALDI‐TOF‐MS: matrix‐assisted laser desorption/ionization ‐time‐ of‐ flight mass spectrometry
MSA: multiple sequence alignment
MWCO: molecular weight cut‐off
PAGE: polyacrylamide gel electrophoresis
PCR: polymerase chain reaction
PDB: protein data bank
pI: isoelectric point
PMSF: phenylmethylsulfonyl fluoride
pNA: para‐nitroanilide
SDS: sodium dodecyl sulfphate
SPAO: subtilisin protease *A. okhensis*
suc: N‐succinyl
TCA: trichloroacetic acid
tet: tetracycline
TFA: trifluoroacetic acid
T_m_
: melting temperature

Proteases are among the most commercially valuable enzymes, with subtilisins or alkaline proteases from microbial sources accounting for the largest market share [1, 2]. They have been extensively studied in terms of their biological function to gain insights into the mechanism of enzyme catalysis and the structure–function relationship of proteins, and because of their significant applications in various industries [1]. Subtilisins belong to the group of subtilases, which is one of the largest families of serine peptidases, and are classified as S8 according to the MEROPS database [3]. Furthermore, subtilisins are further classified as true subtilisins, high‐alkaline subtilisin, intracellular subtilisin, and phylogenetically intermediate subtilisins (PIS) [4, 5]. They are ubiquitously distributed in various organisms, including bacteria, archaea, eukaryotes, yeasts, and viruses [4]. However, *Bacillus* as the most prominent source spawned alkaline proteases such as subtilisin Carlsberg, BPN', and Savinase, with their major application as detergent enzymes with excellent properties including high stability toward extreme temperatures, pH, organic solvents, detergents, and oxidizing compounds [6, 7]. Besides application in detergents, subtilisins find applications, for example, in leather processing, food, wastewater treatment, and cosmetics [6, 8].

The extracellular subtilisins of microorganisms are mainly involved in nutrient supply, and their properties are thought to depend entirely on the host and its adaptability to the immediate environment [6]. Since the size of the microbial world is almost unlimited, and many different microbial sources can be exploited, this biodiversity holds great potential for enriching the repertoire of known enzymes with new and high‐performing enzymes. Enzymes isolated from extremophilic microorganisms such as thermophilic, psychrophilic, and especially halotolerant or halophilic organisms offer enormous potential to meet industrial needs, as evidenced by the increasing number of newly characterized subtilisins [9, 10, 11]. In addition to classical methods of screening microorganisms with new interesting proteases from various environments, different molecular biology techniques, such as metagenomic analysis, directed evolution, and site‐directed mutagenesis have been used to gain or engineer numerous proteases with improved or novel properties [9]. Beside these labor‐intensive methods, genome sequencing and automated annotation are adding potential sequences to the rapidly growing online database and provide an alternative approach to search for candidate protease genes for industrial applications.

*Alkalihalobacillus okhensis* Kh10‐101^T^ is a gram‐positive, strictly aerobic, rod‐like bacterium isolated by Nowlan et al. [12] from an Indian saltpan near the port of Okha. The strain was first classified as *Bacillus okhensis* and in 2020 reclassified into *Alkalihalobacillus okhensis* by Patel and Gupta [13]. As described by Krishna et al. [14], the genome of *A. okhensis* encoded almost 40 different proteases with members of the serine protease family. Since *A. okhensis* is described as a moderate halophile and an alkaliphile with optimal growth conditions of pH 10 and 5% NaCl, the extracellular proteases derived from this organism may be of potential industrial importance [14]. In this research, the gene for subtilisin WP_034632645.1 was cloned, overexpressed in *B. subtilis* DB104, and purified by ion‐exchange chromatography. This is the first report on the biochemical characterization of the recombinant subtilisin protease of *A. okhensis* (SPAO) and includes a comparison with the commercially applied subtilisins Carlsberg, Savinase, and BPN'.

## Materials and methods

### Reagents and enzymes

Polymerase chain reactions (PCR) were performed with Phusion® Hot Start II High‐Fidelity Green Master Mix from Thermo Fisher Scientific GmbH (Karlsruhe, Germany). Oligonucleotides were synthesized by Eurofins Genomics GmbH (Ebersberg, Germany). Restriction enzymes, T4 DNA Ligase, and GeneRuler™ 1 kb DNA ladder were purchased from Thermo Fisher Scientific GmbH. Peptide protease substrates were purchased from BACHEM (Bubendorf, Switzerland). Azocasein, α‐CHCA (alpha‐cyano‐4‐hydroxycinnamic acid), subtilisin Carlsberg, and Savinase were purchased from Sigma‐Aldrich (Schnelldorf, Germany). BPN' was from DuPont (Wilmington, NC, USA). MALDI‐TOF MS protein standards were purchased from LaserBio Labs (Valbonne, France). Lysozyme from chicken and materials for isoelectric focusing (IEF) were purchased from SERVA (Heidelberg, Germany). Centrifugal spin columns and PMSF (phenylmethylsulfonyl fluoride) were purchased from Avantor VWR (Radnor, PA, USA). Molecular weight marker for use with SDS/PAGE (sodium dodecyl sulfate–polyacrylamide gel electrophoresis) was purchased from Bio‐Rad (Hercules, CA, USA). All other chemicals were acquired from Carl Roth (Karlsruhe, Germany).

### Bioinformatic analysis

To illuminate the sequence similarity between different well‐known characterized subtilisins and the protease SPAO, a multiple sequence alignment (MSA) was performed using the mature subtilisin/serine protease sequences from various *Bacillus* strains. The SPAO sequence was blasted by employing the blastp suite of NCBI (https://blast.ncbi.nlm.nih.gov/Blast.cgi). The signal peptide and propeptide sequences were excluded before MSA, and phylogenetic tree construction was performed via Phylogeny.fr (http://www.phylogeny.fr/index.cgi) using the ‘One‐Click’ option [15]. MSA for analysis with ESPript 3.0 was performed with clustal omega (https://www.ebi.ac.uk/Tools/msa/clustalo/) [16, 17]. Phylogenetic trees were displayed with the itol software (https://itol.embl.de/) [18]. MSA was drawn with ESPript 3.0 using %strict option (percentage of strictly conserved residues per column) for the coloring scheme (https://espript.ibcp.fr/ESPript/ESPript/). For homology modeling, the functional amino acid sequence of SPAO without its signal peptide and propeptide was used. A structure prediction was performed through the Iterative Threading Assembly Refinement (i‐tasser) server (https://zhanggroup.org/I‐TASSER/) [19]. The homology model was visualized with Mol* Viewer (https://www.rcsb.org/3d‐view) [20]. Swiss‐PdbViewer (http://www.expasy.org/spdbv/) was used to determine surface‐exposed residues and to calculate the electrostatic potential with standard settings using the Poisson–Boltzmann Equation [21]. The theoretical pI and molecular mass of the functional domain were calculated with https://web.expasy.org/compute_pi/. The identification of the signal peptide was performed with the signalp6.0 software https://services.healthtech.dtu.dk/service.php?SignalP‐6.0 [22].

### Strains and growth conditions

*Alkalihalobacillus okhensis* Kh10‐101^T^ (DSM 23308) was purchased from the German collection of microorganisms and cell cultures GmbH (DSMZ) and cultivated according to their recommendations in medium 830, pH 9.5, at 35 °C. An overnight culture was used for genomic DNA preparation with the InnuSPEED Bacteria/Fungi DNA Kit (Analytik Jena™, Jena, Germany) according to the manufacturer's recommendations. *Bacillus subtilis* DB104 was used as host for cloning and protein production [23] and cultivated in lysogeny broth (LB) medium (10 g·L^−1^ tryptone, 5 g·L^−1^ yeast extract, 10 g·L^−1^ NaCl, pH 7.0; Carl Roth).

### Plasmid construction and cloning

A pBC16‐based expression plasmid (Acct. No. U32369.1) was used for recombinant protease production with *B. subtilis* DB104 [24]. The pBC16 derivative pFF‐RED was obtained by exchanging the *mob* region by an expression cassette comprising the subtilisin Carlsberg promoter from *B. paralicheniformis* ATCC 9945a followed by the gene *eforRED* encoding a red chromoprotein (acc. no. ACD13196.1). This marker gene is flanked by BbsI restriction sites allowing cloning the gene of interest via Golden Gate cloning.

The DNA sequences encoding the protease signal peptide, the propeptide, and the mature domain were amplified from the genomic DNA using the Phusion® Hot Start II High‐Fidelity polymerase according to the manufacturer's recommendations. The NCBI reference sequence NZ_JRJU01000039.1 was used to design primers for the *aprE* gene (extracellular alkaline protease) encoding the protein WP_034632645.1 [12]. To remove an internal BbsI restriction site within the propeptide, two PCRs were performed with primers introducing a silent point mutation at the internal BbsI restriction site. For PCR 1, the forward primer (5′‐AAAGAAGACGGAATGAAAAAGTTATTTACGAAAGTAGTTGCC‐3′) and the reverse primer (5′‐GGTTAAAAATACTAACCTCAATATCTTCCTCGATGAAAGCAATAG‐3′) were used. For PCR 2, the forward primer (5′‐CTATTGCTTTCATCGAGGAAGATATTGAGGTTAGTATTTTTAACC‐3′) and the reverse primer (5′‐AAAGAAGACCCGTTATCTTGTAGCAGCTTCGGCATTAACAAG‐3′) were used. The two PCR fragments of 342 and 851 bp were combined within an overlap extension PCR using the two outer primer pairs introducing two BbsI restriction sites and corresponding overhangs to the cloning site of pFF‐RED.

The PCR product was cloned via Golden Gate cloning into the BbsI site of pFF‐RED [25]. The product (pFF003) was subsequently used to transform *B. subtilis* DB104 naturally competent cells as described elsewhere [26].

PCR success was verified by agarose gel electrophoresis. After transformation, clones were spread onto LB agar plates (1.5% (w/v) agar) supplemented with 20 μg·mL^−1^ tetracycline (tet_20_) and 2.5% (w/v) skim milk powder. The clones were grown at 37 °C overnight. Colonies with clear halo zones indicated protease activity and were selected as positive clones. The clones were subsequently cultivated overnight at 37 °C and 200 r.p.m. in 10 mL LB_tet20_ medium. Plasmids from each protease‐positive clone were isolated by GeneJET Plasmid Miniprep Kit (Thermo Fisher Scientific), and the desired cloning result was confirmed by double‐restriction digestion and plasmid DNA Sanger sequencing (Eurofins Genomics).

### Recombinant protease production

Production of the protease by *Bacillus subtilis* DB104 was carried out by inoculating 10 mL of LB_tet20_ medium with a freshly plated clone and cultivated over 8 h at 180 r.p.m. and 37 °C. Subsequently, an overnight culture with 50 mL of the preculture medium (Tables S1 and S3) was inoculated with 100 μL of the over‐the‐day culture. After cultivation overnight at 37 °C and 180 r.p.m., the bioreactors were inoculated to an optical density at 600 nm (OD600) of 0.25. The fermentation was performed in a DASGIP® parallel reactor system (DASGIP, Jülich, Germany) with four 1‐L reactors. The air supply was performed with an L‐sparger and a volume flow of 0.5 vvm. The oxygen saturation was set at 30% and regulated by the stirrer speed (max. 1500 r.p.m., min. 350 r.p.m.). The pH was adjusted to 7.4 and regulated by adding either 4 m NaOH or 20% (v/v) H_2_SO_4_. The reaction temperature was set at 37 °C. For cultivation, a high protein content medium with soy peptone was used (Tables S2 and S3). After 12 h of cultivation, a glucose feed (1.5 g·mL^−1^) was started with 1 mL·h^−1^ for 7 h, then 1.5 mL·h^−1^ for 9 h and 1.1 mL·h^−1^ until the end of the fermentation. After 10 h, a polypropylene glycol 2000 (PPG) feed (0.5 mol·L^−1^) was established for 10 h with 0.25 mL·h^−1^. The fermentation was performed over 48 h, and the supernatant was harvested by centrifugation at 3000 ***g*** for 20 min. For storage at 4 °C, 10% (v/v) of propylene glycol was added to the supernatant. The protease production was confirmed by a proteolytic activity assay using suc‐AAPF‐pNA as substrate and by SDS/PAGE.

### Enzyme purification

One hundred and forty millilitre of the cell‐free supernatant was divided into portions of 15 mL to which 30 mL ice‐cold 96% (v/v) ethanol was added, and the mixture was incubated overnight at −20 °C. After incubation, the samples were centrifuged for 20 min at 13 000 ***g*** and 4 °C. The supernatant was discarded, and the pellets were washed with 15 mL ice‐cold 96% (v/v) EtOH by vortexing. The samples were centrifuged again as described above, and the supernatant was removed. The pellets were resuspended in 7 mL of running buffer (10 mm HEPES/NaOH buffer, pH 8.0). The concentrated sample was then centrifuged as described above before being applied to a 50 mL HiPrep 26/10 desalting column coupled to an Äkta Avant 25 (Cytiva Europe, Freiburg, Germany). The protein was eluted with running buffer. The desalting process was monitored by measuring the conductivity and the absorbance at 280 nm. The peak protein fractions without salt were collected and used for ion‐exchange chromatography.

According to the theoretical isoelectric points and the pH of the buffer used, a cation exchanger column (22 mL S‐Sepharose FF, GE Healthcare, Chicago, IL, USA) was used using the Äkta Avant 25 device (Cytiva Europe GmbH, Freiburg, Germany). The column was equilibrated with running buffer. The desalted protein sample was applied to the ion exchanger column and washed with two column volumes of running buffer. The protease was eluted with a linear salt gradient using the elution buffer (10 mm HEPES/NaOH, pH 8.0, 1 m NaCl) and was collected in fractions of 5 mL. Protein elution was monitored by measuring the absorbance at 280 nm. The protein‐containing fractions were subsequently used for further analysis. For long‐term storage, the purified proteases were stored at −80 °C with 10% (v/v) of glycerol.

### Enzyme activity assay

The hydrolytic activity of proteases was determined with the tetrapeptide substrate *N*‐succinyl‐Ala‐Ala‐Pro‐Phe‐p‐nitroanilide (suc‐AAPF‐pNA) at 30 °C in 100 mm Tris/HCl buffer, pH 8.6, containing 0.1% (w/v) Brij®35 [27]. The substrate was prepared as a 110 mm stock solution in dimethyl sulfoxide and diluted 1 : 100 in the reaction mix. The amount of released *p*‐nitroaniline (Ԑ_410 nm_ = 8.48 mm ^−1^·cm^−1^) was determined by measuring the absorbance at 410 nm for 5 min every 30 s [27]. The reaction was performed either in 1 mL cuvette format (Ultrospec 2100 pro, Amersham Biosciences, Little Chalfont, UK) or in microtiter format (Infinite 200Pro, Tecan, Männedorf, Switzerland) with a reaction volume of 1 mL and 250 μL, respectively. One unit (U) of enzyme activity was defined as the amount of enzyme that produced 1 μmol of *p*‐nitroaniline per minute under the assay conditions.

Protease activity was determined using azocasein as a substrate based on Brock et al. [28]. The partial hydrolysis of the substrate by the proteases releases dye‐labeled smaller peptides that are no longer precipitable. The dye‐labeled peptides were quantified at a wavelength of 440 nm. For the assay, a fresh substrate solution of 2% (w/v) azocasein in 100 mm Tris/HCl, pH 8.6, was prepared. The reaction was performed in 125 μL substrate solution with 75 μL of the protease sample in an appropriate dilution. The mixture was incubated at 37 °C and 300 r.p.m. for exact 30 min. The reaction was stopped by adding 600 μL of 20% (w/v) trichloroacetic acid (TCA) and kept at room temperature for 15 min, followed by centrifugation at 12 500 ***g*** for 5 min. Then, 600 μL of the supernatant was mixed with 700 μL of 1 m NaOH, and the absorbance at 440 nm (0.5 ± 0.1) was measured. One unit (U) of activity was defined as the amount of enzyme required to increase the corresponding absorbance value by 0.01 units per minute under the conditions described above.

### Protein measurement, electrophoresis, and analytical methods

Protein concentration was determined by measuring the absorbance ratio 590/450 nm using Roti® Nanoquant (Carl Roth) with bovine serum albumin fraction V (Carl Roth) as a standard based on the method of Bradford [29].

The molecular mass of the purified proteases was analyzed by matrix‐assisted laser desorption/ionization time‐of‐flight mass spectrometry (MALDI‐TOF‐MS) using an Axima confidence (Shimadzu Europe, Duisburg, Germany) in linear positive mode with pulsed extraction optimized for the theoretical molecular mass. Data were analyzed with mmass [30]. Purified protease was precipitated by mixing 1 : 2 with 20% (w/v) TCA. The precipitate was resuspended with 50 μL dH_2_O/0.1% (v/v) trifluoroacetic acid (TFA) and 2 μL of 2 m NaOH. Samples were diluted 1 : 10 with α‐cyano‐4‐hydroxycinnamic acid (CHCA; Sigma‐Aldrich) as matrix. One microlitre of the dilution was applied onto the MALDI‐TOF‐MS target plate and air‐dried, and then, again 1 μL was applied. As mass standards, trypsinogen and bovine serum albumin from LaserBio (Valbonne, France) were used.

SDS/PAGE was performed using an 8–20% (v/v) resolving gel and a 6% (v/v) stacking gel as described by Miller et al. [31]. For sample preparation, 50 μL of 20% (w/v) TCA was added to 25 μL of the protein sample. The mixture was incubated for 2 min on ice and then centrifuged for 5 min at 12 000 ***g***. The supernatant was carefully discarded, and 50 μL of 2 × reducing SDS sample buffer after Laemmli and 50 μL of 0.1 m NaOH were added to the precipitated protein. The sample was subsequently boiled for 10 min at 95 °C. The electrophoresis was performed for 30 min at 300 V in Tris‐glycine/SDS (Laemmli) buffer, pH 8.6. The gel was stained with Roti® Blue quick (Carl Roth) for 2 h, destained in dH_2_O for 30 min, and photographed with the UVP® GelStudio touch device (Analytic Jena, Jena, Germany).

For IEF‐PAGE, purified SPAO and the reference proteases were rebuffered in 10 mm HEPES/NaOH, pH 7.0, using centrifugal spin columns with a molecular weight cutoff (MWCO) of 3 kDa. The SERVAGel™ IEF 3–10 gels were used according to the manufacturer's recommendations.

### Effect of sodium dodecyl sulfate, hydrogen peroxide, and phenylmethylsulfonyl fluoride (PMSF) on enzyme activity and stability

To examine the effect of surfactant and oxidizing agents, hydrogen peroxide (1 and 5% (v/v)) and SDS (1 and 5% (w/v)) were added to the enzyme solution in 10 mm HEPES/NaOH, pH 8.0, and incubated for 1 h at 10 °C. The low incubation temperature was chosen to prevent autoproteolysis. The remaining activity was measured in the standard suc‐AAPF‐pNA activity assay. Residual activity of the proteases incubated in buffer with no additives was set as 100%.

The effect of 1 mm of the protease inhibitor PMSF was investigated by incubating the proteases in 10 mm HEPES/NaOH, pH 8.0, for 30 min on ice. Residual activity of the proteases incubated in buffer with no additions was set as 100%.

### Effect of NaCl, EDTA, and CaCl_2_ on enzyme activity and stability

The effect of NaCl on proteolytic activity was measured under standard reaction conditions for suc‐AAPF‐pNA with NaCl (0–5 m) in the reaction buffer. The influence of NaCl on enzyme stability was investigated by incubating the proteases in 10 mm HEPES/NaOH, pH 8.0, with NaCl (0–5 m) at 20 °C for 2 h. The % residual activities measured before incubation were set as 100%.

For the investigation of the effect of ethylenediaminetetraacetic acid (EDTA) and CaCl_2,_ the purified proteases were rebuffered in centrifugal spin columns (3 kDa MWCO). 10 mM HEPES/NaOH, pH 8.0, was added twice to the retentate after centrifugation at 4 °C and 12 000 ***g***. The rebuffered proteases (20 μg·mL^−1^) were incubated for 12 h at 4 °C with and without 20 mm EDTA in duplicates. After incubation, the reaction mixtures were diluted properly for analysis in a modified suc‐AAPF‐pNA assay. In this case, the diluted protease sample was incubated with reaction buffer containing CaCl_2_ (0–25 mm) for 5 min before adding the substrate solution (suc‐AAPF‐pNA, 1.1 mm final).

### Substrate spectrum

The substrate specificity of the proteases was determined using the synthetic peptide‐4‐nitroanilide substrates suc‐Tyr‐Val‐Ala‐Asp‐pNA (YVAD), suc‐Phe‐Ala‐Ala‐Phe‐pNA (FAAF), suc‐Ala‐Ala‐Ala‐pNA (AAA), suc‐Ala‐Ala‐Val‐Ala‐pNA (AAVA), suc‐Ala‐Leu‐Pro‐Phe‐pNA (ALPF), suc‐Ala‐Gly‐Pro‐Phe‐pNA (AGPF), suc‐Ala‐Ala‐Pro‐Phe‐pNA (AAPF), suc‐Ala‐Ala‐Pro‐Leu‐pNA (AAPL), suc‐Thr‐Val‐Ala‐Ala‐pNA (TVAA), and suc‐Ala‐Gly‐Pro‐Pro‐pNA (AGPP) dissolved in dimethyl sulfoxide to a final concentration of 0.34 mm in the assay. Kinetic experiments were carried out as described above.

### Effects of temperature and pH on enzyme activity and stability

To monitor thermal protein unfolding and to determine the melting point of the proteases, the environmentally sensitive fluorescent dye SYPRO™ Orange (Thermo Fisher Scientific GmbH) was used. During the unfolding process at higher temperatures, hydrophobic residues get exposed causing an increase in the SYPRO™ Orange fluorescence, which is monitored (Ex/Em = 470/550 nm) [32]. The unfolding kinetics were performed in a qPCR cycler (qTower3G, Analytic Jena) in at least triplicates. The detection mixture contained 5 × SYPRO™ Orange solution in 10 mm HEPES/NaOH buffer, pH 8.0, with 3 mm PMSF and purified and rebuffered proteases (35–320 μg·μL^−1^). The purified proteases were rebuffered in centrifugal spin columns (3 kDa MWCO). 10 mm HEPES/NaOH, pH 8.0, was added twice to the retentate after centrifugation at 4 °C and 12 000 ***g***. The thermocycler block was heated from 25 to 95 °C in steps of 2 °C with a 2‐min holding time measuring fluorescence intensities at each step. The data analysis was performed using qpcrsoft 4.0 (Analytic Jena), and apparent T_m_ values were calculated for each protease as the inflection point of the melting curve.

The temperature optimum was assayed between 20 and 90 °C in 5 °C steps with the suc‐AAPF‐pNA assay as described above. Before every measurement, the cuvette holder and the buffer were preheated at least for 5 min at the desired temperature. The pH of the reaction buffer was adapted until 80 °C. For each measurement, a new blank was used to exclude thermal instability of the substrate. The temperature stability was determined by measuring the residual activity after incubating the enzymes in 10 mm HEPES/NaOH, pH 8.0, at 20 and 50 °C for 3 h. The % residual activities were followed by measuring the residual activities every 20 min under standard reaction conditions for the suc‐AAPF‐pNA assay.

The optimal pH of the proteases was determined at 30 °C in 0.1 m Tris/maleate buffer (pH 5.0–7.0), 0.1 m Tris/HCl (pH 7.0–9.0) and 0.1 m glycine/NaOH (pH 9.0–12.5) under standard reaction conditions for suc‐AAPF‐pNA substrate. The effect of pH on enzyme stability was assayed by preincubating enzymes in said buffers for 4 h at 4 °C to prevent autoproteolysis. The % residual activities were measured under standard reaction conditions for the suc‐AAPF‐pNA assay.

## Results and discussion

### Cloning and expression of *aprE_A. okhensis* in *B. subtilis* DB104

The gene sequence of *aprE_A. okhensis* for SPAO was amplified from the halophilic strain *A. okhensis* Kh10‐101^T^ as described in Materials and methods. A fragment of 1148 bp containing the coding region for the signal peptide, the propeptide, and the mature part of the protease was obtained, cloned into the vector pFF‐RED, and transferred into *B. subtilis* DB104. Transformants with a clearing zone on LB agar plates supplemented with 2.5% (w/v) skim milk powder were analyzed by plasmid preparation, and pFF003 coding for SPAO was identified by restriction digestion‐based molecular screening. Sequence data obtained by the Sanger sequencing method showed that the inserted sequence was identical to the nucleotide sequence of the *aprE_A. okhensis* gene available in GenBank, except for the elimination of the internal BbsI site in the coding region of the propeptide.

### Bioinformatic analysis and homology modeling

The *aprE* gene from *A. okhensis* Kh10‐101^T^ comprises 1149 bp encoding a protein of 382 amino acids (aa). Most subtilases have a multi‐domain structure consisting of a signal peptide (for translocation), a propeptide (for maturation by autoproteolytic cleavage), a protease domain, and frequently one or more additional domains [33]. The signal peptide prediction revealed a Sec signal peptide with a cleavage site between amino acids 27 and 28 with a probability of 96.9% [22]. The propeptide was identified by multiple sequence alignment to comprise amino acids 29–113. Thus, three functional domains were identified with a 27‐aa signal peptide, a 86‐aa propeptide, and a 269‐aa mature endopeptidase S8 domain (Fig. 2). The analysis of the mature protease sequence revealed a molecular mass of 27.14 kDa and a calculated pI of 9.6. The catalytic triad consisted of Asp^32^, His^62^, and Ser^215^ (numbers based on the mature protease sequence).

**Fig. 1:**
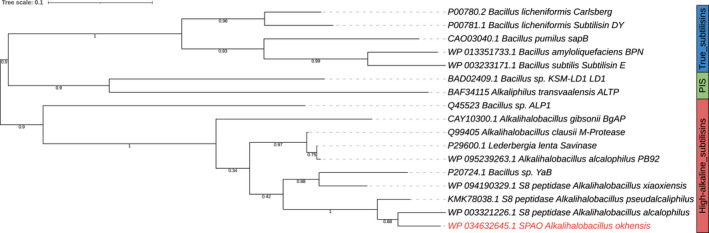
Evolutionary phylogenetic tree of various subtilisins from different species of the family *Bacillaceae*. Maximum‐likelihood phylogenetic analysis of the mature protease domains was performed using the Phylogeny.fr server. Numbers at nodes indicate support for the internal branches within the tree obtained by approximate likelihood ratio test (SH‐like aLRT).

**Fig. 2:**
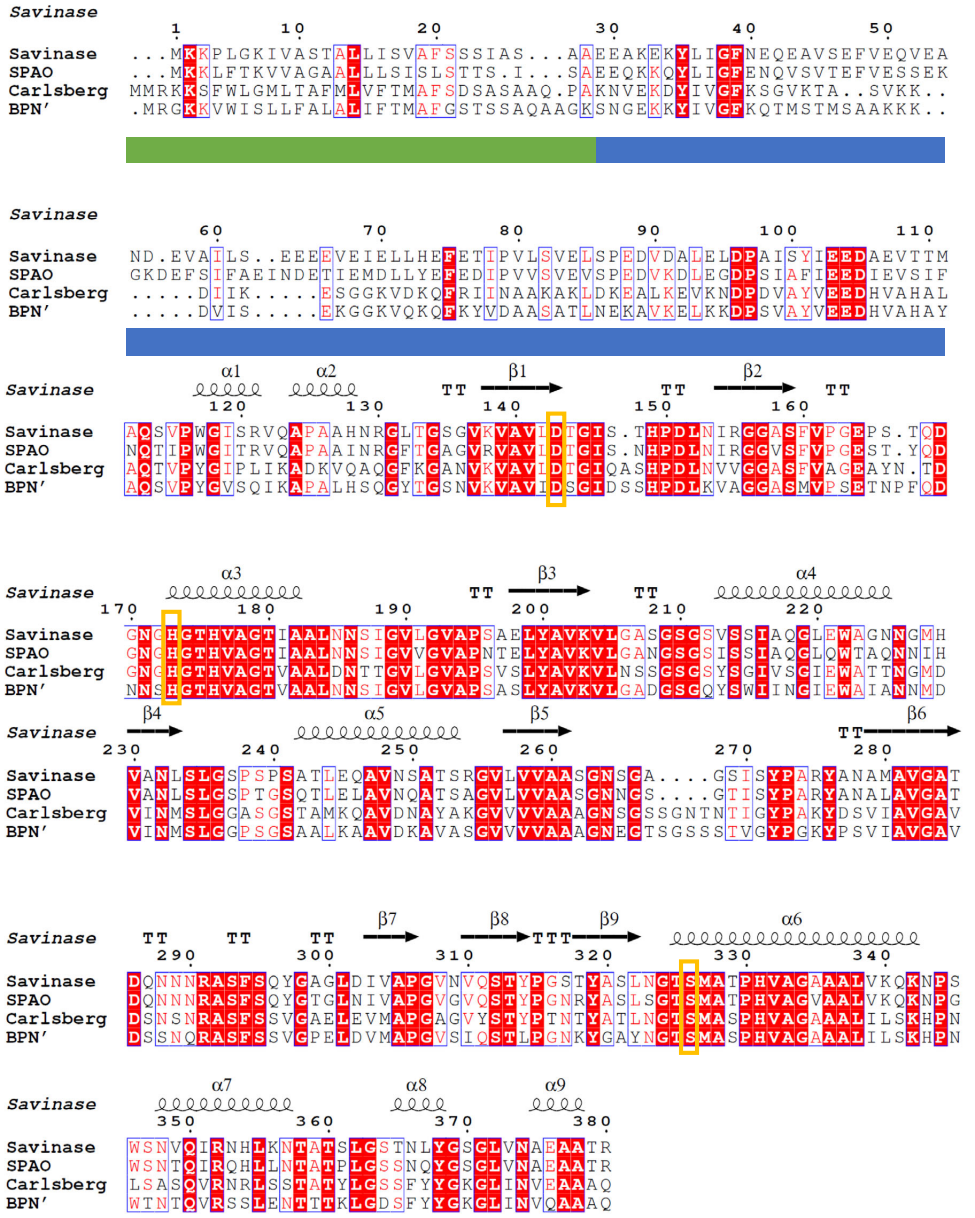
Multiple sequence alignment (MSA) of SPAO with Savinase (WP_094423791.1), subtilisin Carlsberg (WP_020450819.1), and BPN' (WP_013351733.1). The alignment was calculated by clustal omega and drawn using ESPript 3.0 and Savinase (PDB: 1C9J) as a template. Solid green and blue bars indicate the signal peptide sequence and propeptide of SPAO. Secondary structure elements are presented on top (helices with squiggles, β‐strands with arrows, and turns with TT letters). Orange boxes show residues comprising the catalytic triad (Asp^145^, His^175^, Ser^328^; SPAO numbering).

The predicted amino acid sequence of the mature part of SPAO was aligned and compared with well‐characterized proteases of the three subtilisin families (true, high‐alkaline, phylogenetic intermediate) retrieved from MEROPS [3] and the UniProt database [34] resulting in a phylogenetic tree (Fig. 1). Additionally, a BlastP search was performed. The mature part of SPAO showed the highest sequence similarity with a subtilisin from *Alkalihalobacillus alcalophilus* (91.45%), which corresponds to subtilisin Sendai from *Bacillus sp*. G‐825‐6 [35]. Furthermore, it showed high similarity to the subtilisin of *Alkalihalobacillus pseudoalkaliphilus* (90.33%), subtilisin Savinase from *Lederbergia lenta* (formerly *Bacillus lentus*; 82.44%) [36], and subtilisin PB92 from *Alkalihalobacillus alcalophilus* (82.84%) [37]. Moreover, SPAO showed a more distant relationship to the well‐characterized true subtilisins BPN' (56.13%) [38] and subtilisin Carlsberg (57.84%) [39]. In the phylogenetic tree, SPAO is therefore clearly distinct from the phylogenetic intermediate subtilisins and the true subtilisins, indicating its affiliation with the highly alkaline subtilisin group. Therefore, for comparative biochemical characterization, the two true subtilisins BPN' and subtilisin Carlsberg and the high‐alkaline subtilisin Savinase were used. The MSA for those three enzymes and SPAO is shown in Fig. 2. The sequence similarity between SPAO and Savinase is high as mentioned above and shows typical amino acid residues for high‐alkaline subtilisins, which were identified by MSA of true subtilisins, PIS, and highly alkaline subtilisins, as described by Yamagata et al. [40].

**Fig. 3:**
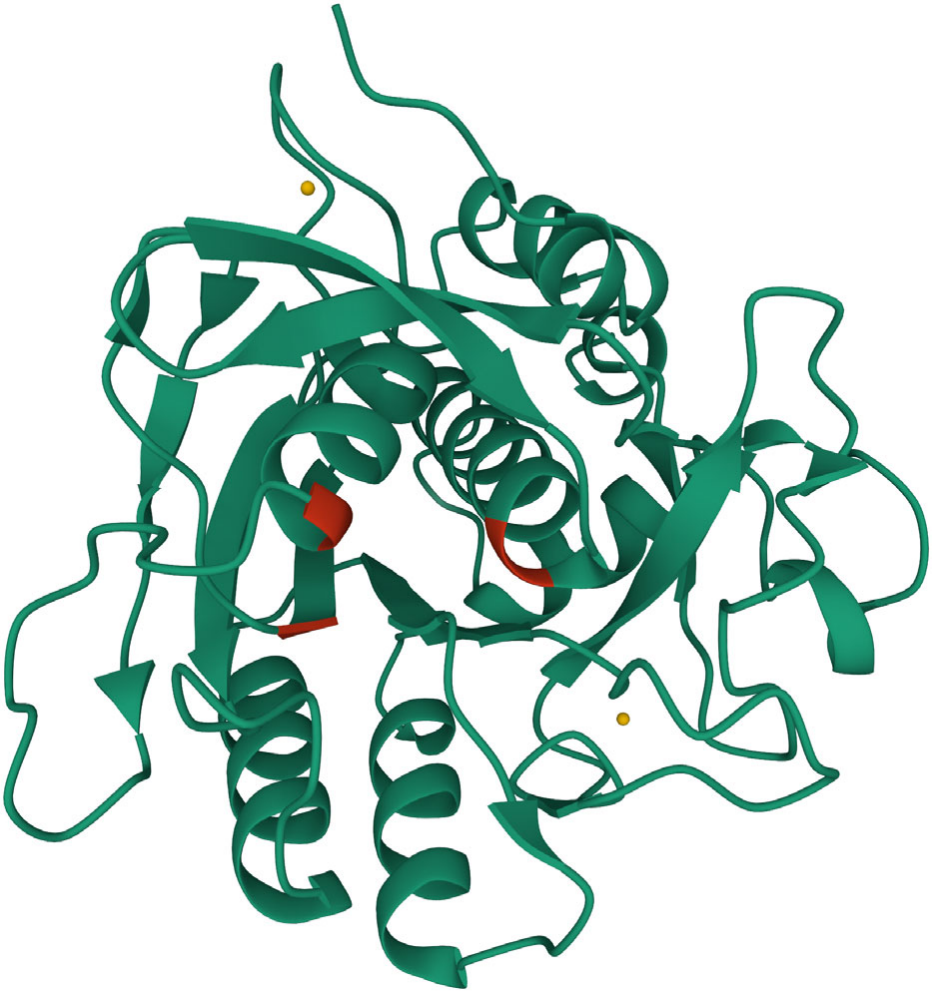
Homology model of mature SPAO using i‐tasser software. *In silico* metal‐binding analysis predicted the existence of two Ca^2+^ binding sites (yellow balls). The catalytic residues Asp^32^, His^62^, and Ser^215^ are shown in red.

**Fig. 4:**
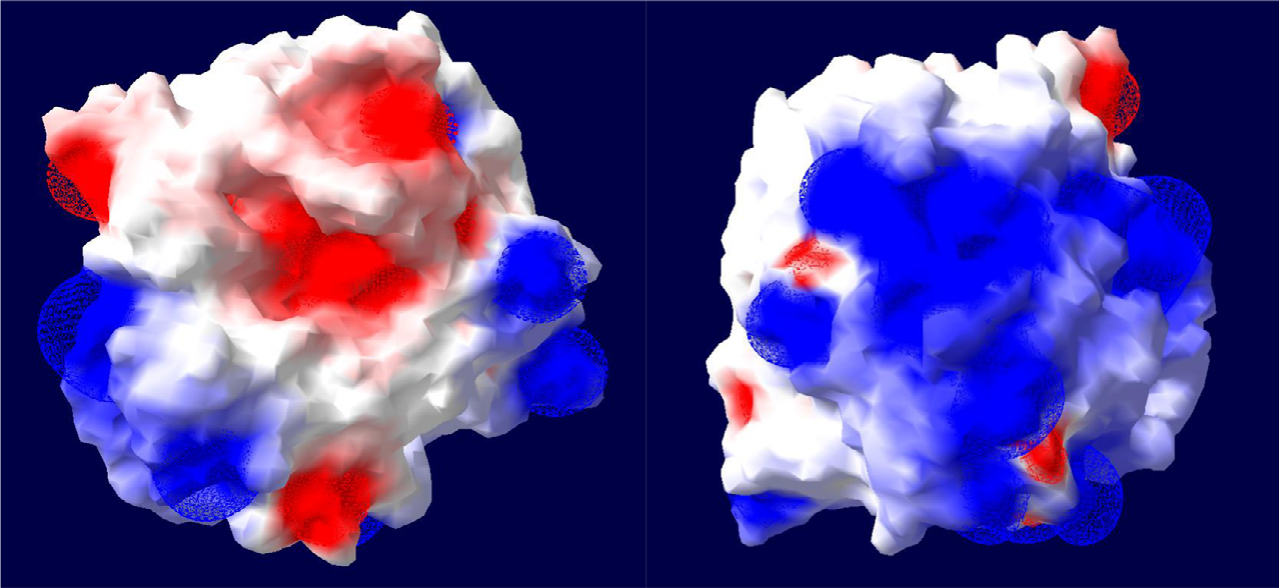
Protein surface electrostatic potential calculations for the structural model of SPAO. (Left) front view of the active site; (right) back of the active site. Electrostatic potential at pH 7.0 is shown as red (negative) and blue (positive) and was calculated using the Swiss‐PdbViewer.

The 3D structure identification of an enzyme is of great interest in order to identify potential key residues essential for the specific activity, halotolerance, etc. These key residues and their interactions also play a role in potential enzyme engineering efforts. Therefore, homology modeling‐based structural analysis of recombinant SPAO was also performed. The predicted 3D structure of mature SPAO calculated with the i‐tasser server [19] is shown in Fig. 3 with a C‐score of 1.55. The C‐score measurement determines the quality of the resulting models in the range [−5.2], where a C‐score with a higher value means a model with high confidence [41]. The highest structural similarity (99.4%) was displayed to *Lederbergia lenta* subtilisin Savinase (PDB: 1C9J) with a template modeling score of 0.994 and 0.46 Å root‐mean‐square deviation. In general, subtilisins show a great similarity in their molecular structure [42]. *In silico* analysis of the model for probable metal‐binding sites suggested that SPAO harbors two potential Ca^2+^ binding sites within its functional domain (site 1: Asp^40^, Leu^73^, Val^79^, Ile^77^; site 2: Ala^163^, Ala^168^). Using this model to analyze the surface‐exposed residues with the Swiss‐PdbViewer at a threshold of 20% of the accessible surface revealed that six Arg residues and one Lys residue are exposed at the surface, which is important for salt and pH adaptation, as will be explained later [21]. In addition, the electrostatic potential of SPAO was evaluated using the Swiss‐PdbViewer and showed a mainly positively charged backside and a negatively charged region around the active site at pH 7.0, which helps to interpret the adaption to high salt concentrations as discussed later (Fig. 4).

### Recombinant protease production and purification

**Fig. 5:**
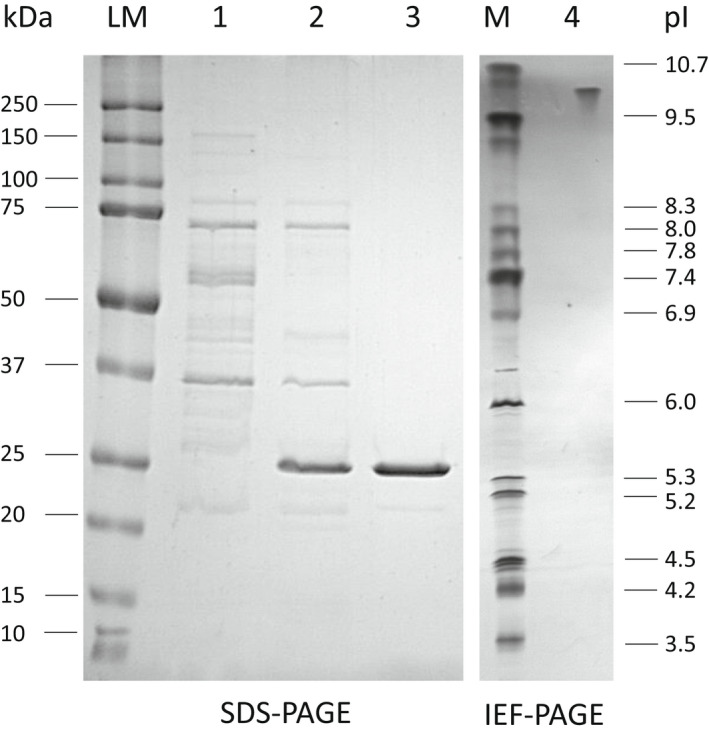
SDS/PAGE and IEF‐PAGE analysis of recombinant SPAO. Samples were electrophoresed using an 8–20% SDS/polyacrylamide gel. As protein standards, the LM mixture Bio‐Rad Precision Plus Dual Color was used. Culture supernatant of *B. subtilis* DB104 carrying pFF‐RED (lane 1); culture supernatant of *B. subtilis* DB104 carrying pFF003 producing SPAO (lane 2); and SPAO after purification (lane 3). Purified and desalted SPAO on an IEF gel SERVALYT™ PRECOTES™ wide range pH 3–10 (lane 4) and marker (M; SERVA IEF marker 3–10).

SPAO was produced by *B. subtilis* DB104 pFF003 at 1‐L scale using a DASGIP parallel fermentation device as described in Materials and methods. The culture supernatant showed an activity of 103 U·mL^−1^ (AAPF) and a protein concentration of 0.6 mg·mL^−1^. It was used for a three‐step purification process that involved ethanol precipitation, desalting, and ion‐exchange chromatography. Successful production and apparent homogeneity of SPAO were confirmed via SDS/PAGE (Fig. 5). The purified SPAO had a molecular mass of about 27 kDa on SDS/PAGE, which correlates to the theoretical mass of 27.1 kDa (Fig. 5). Additionally, the size of 27.1 kDa was confirmed by MALDI‐TOF MS analysis (Fig. S1). The purified protease had a specific activity of 139 U·mg^−1^ for the AAPF substrate and 528 U·mg^−1^ for azocasein. Similar activities after production with *B. subtilis* and purification could be achieved for subtilisin E with 486 U·mg^−1^ for azocasein under the same experimental conditions [43]. The analysis of the purified and rebuffered SPAO for its isoelectric point revealed a pI of approx. 9.8, which is close to the predicted pI of 9.6 (Fig. 5). Subtilisin Carlsberg showed a pI of 8.0 and Savinase of 9.8 (data not shown). High‐alkaline subtilisins have a high pI in common [44].

### Effects of temperature and pH on enzyme activity and stability

**Fig. 6:**
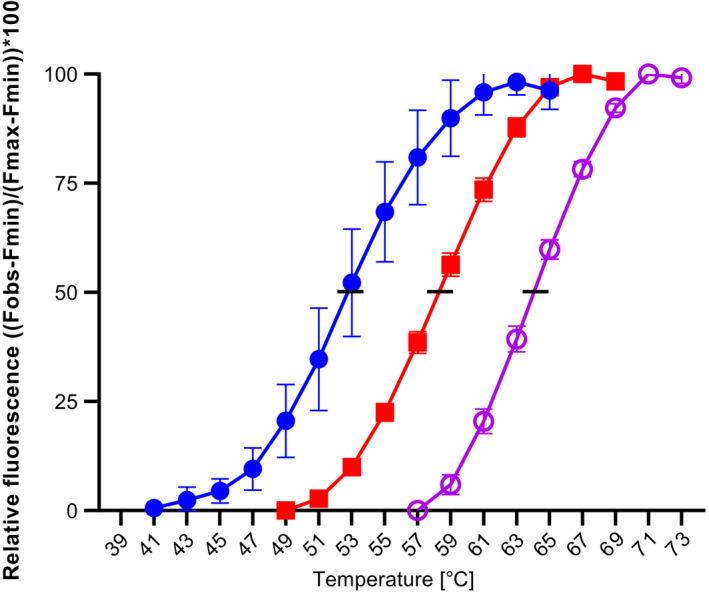
Melting curves of purified SPAO in comparison with BPN' and subtilisin Carlsberg. The effect of temperature on the stability of the enzyme using SYPRO® Orange as a fluorescence probe, based on the changes in fluorescence emission intensity (Ex/Em = 470/550 nm; 5 × SYPRO® Orange, 10 mm HEPES/NaOH, pH 8.0, 3 mm PMSF), is shown as normalized denaturation curves of the thermal shift assay for the proteases SPAO (closed circles), BPN' (squares), and subtilisin Carlsberg (open circles). The inflection point corresponds to the melting temperature (T_m_), at which 50% of the protein is unfolded (−). The experiment was performed in triplicates, and data are plotted as mean values ± SD.

**Fig. 7:**
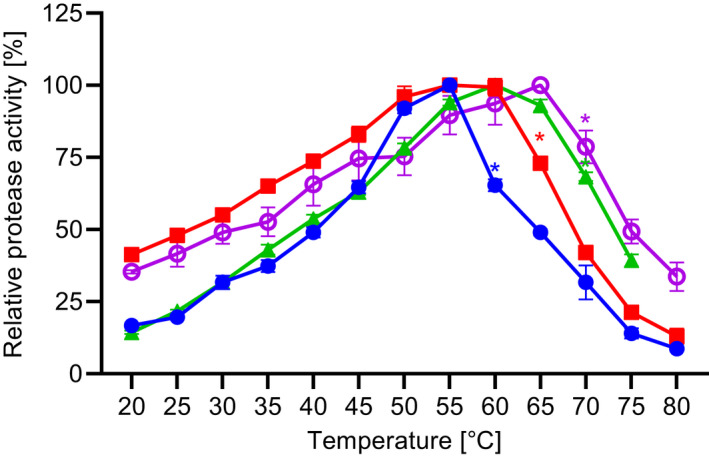
Effect of temperature on the activity of purified SPAO, BPN', Savinase, and subtilisin Carlsberg. The activities of the proteases were determined by assaying protease activity at temperatures between 20 and 90 °C with suc‐AAPF‐pNA assay. The maximum activity for each protease was considered as 100% activity; SPAO (closed circles; 1076 U·mg^−1^), BPN' (squares; 1007 U·mg^−1^), Savinase (triangles; 2264 U·mg^−1^), and subtilisin Carlsberg (open circles; 2767 U·mg^−1^). *The enzyme was not stable for the intended 5 min. The experiment was performed in triplicates, and data are plotted as mean values ± SD.

Stability measurements of proteolytic enzymes are difficult due to potential autoproteolytic cleavage during unfolding. To monitor thermal protein unfolding rather than autoproteolysis and to obtain an estimate of the conformational stability of SPAO, the denaturation curve of the enzyme inhibited by PMSF was measured. The inhibition with 1 mm PMSF was tested before, resulting in a complete loss of activity (Table 1). Figure 6 shows the normalized fluorescent signal curves (relative to the maximal fluorescence measured) for SPAO in comparison with the well‐characterized proteases BPN' and subtilisin Carlsberg. SPAO showed a T_m_ value of 53.0 °C, which was lower than the T_m_ values of BPN´ (58.5 °C) and subtilisin Carlsberg (64.0 °C). As expected, the proteases having a higher temperature optimum (see below; Fig. 7) display also a higher melting point, suggesting higher structural integrity at elevated temperatures. In applications such as detergents, the trend is toward more cold‐active enzymes, which, however, lack stability at higher temperatures [32, 45]. The high‐alkaline subtilisin BgAP from *Bacillus gibsonii* showed a melting point of 52.5 °C in the thermal shift assay comparable to SPAO, while several rounds of sequence saturation mutagenesis increased thermal stability to 58 °C with an additional reduction in the temperature optimum [32].

**Table 1:** Influence of H_2_O_2_, SDS, and PMSF on enzyme activity. The purified proteases were incubated with 1 and 5% (w/v) SDS; 1 and 5% (v/v) H_2_O_2_; and 1 mm PMSF at 10 °C in 10 mm HEPES/NaOH pH 7.0 for 1 h. Residual activity of the proteases incubated in buffer with no additions was considered as 100%. All experiments were performed at least in triplicates, and data are shown as mean values ± SD.

Protease	Residual protease activity [%]				
	1% SDS	5% SDS	1% H_2_O_2_	5% H_2_O_2_	1 mm PMSF
SPAO	0 ± 0	0 ± 0	108 ± 4	58 ± 3	0 ± 0
Subtilisin Carlsberg	325 ± 6	189 ± 6	71 ± 9	27 ± 2	0 ± 0
Savinase	138 ± 4	113 ± 6	64 ± 3	8 ± 0	0 ± 0
BPN'	205 ± 3	164 ± 7	81 ± 5	11 ± 0	0 ± 0

The effect of temperature on the enzyme activity was studied in a temperature range of 20–90 °C at pH 8.6 (standard suc‐AAPF‐pNA assay), as shown in Fig. 7. SPAO showed a temperature optimum of 55 °C, while BPN', Savinase, and subtilisin Carlsberg exhibited temperature optima of 55, 60, and 65 °C, respectively. The lower temperature optimum of SPAO correlates with temperature growth rates of the bacterial origin (25–40 °C) [12]. For subtilisin Carlsberg, the higher temperature optimum coincides with the growth optimum of mesophilic *Bacillus licheniformis*. A serine protease isolated from the thermophilic *Geobacillus toebii* LBT 77 elevated an even higher optimum of 95 °C [46], while most other serine proteases from *Bacilli* have a temperature optimum between 50 and 70 °C [47, 48, 49, 50].

**Fig. 8:**
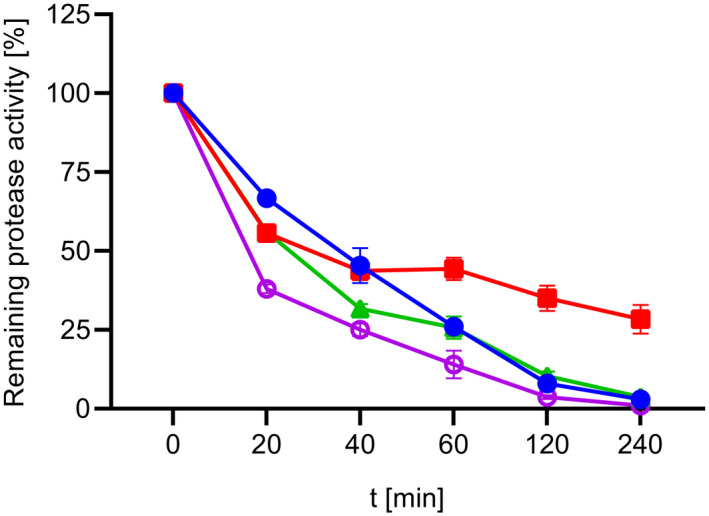
Temperature stability of purified SPAO, BPN', Savinase, and subtilisin Carlsberg. Stability was investigated at 20 °C in 10 mm HEPES/NaOH buffer, pH 8.0. The activity was measured with the suc‐AAPF‐pNA assay in reaction buffer at 30 °C. The activity at 0 min was considered as 100% activity; SPAO (closed circles; 325 U·mg^−1^), BPN' (squares; 343 U·mg^−1^), Savinase (triangles; 367 U·mg^−1^), and subtilisin Carlsberg (open circles; 635 U·mg^−1^). The experiment was performed in triplicates, and data are plotted as mean values ± SD.

Figure 8 shows the loss of protease activity during incubation for 4 h at 20 °C. SPAO showed a similar loss of activity as Savinase and subtilisin Carlsberg, with almost no activity remaining after 4 h, while BPN' retained about one‐third of its activity after this time. As expected, the loss of activity is more pronounced for all proteases when incubated at 50 °C with residual activities of less than 25% (data not shown). Phrommao et al. [51] reported high stability at 20 °C but complete loss of activity after 2 h of incubation at 50 °C for the alkaline serine protease from *Virgibacillus sp*. SK37. The subtilisin from *Bacillus halodurans* C‐125 demonstrated high stability with 94% remaining activity after 1 h of incubation at 50 °C [52]. A comparable loss of activity for subtilisin Carlsberg was also observed by others [53, 54]. Higher thermal stability could be achieved by the addition of stabilizers such as polyols, which hindered the unfolding process and therefore reduced autoproteolysis [55, 56].

**Fig. 9:**
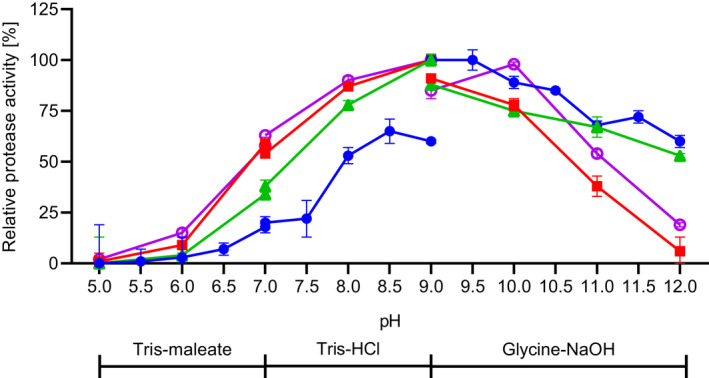
Effect of pH on the activity of purified SPAO, BPN', Savinase, and subtilisin Carlsberg. The activity was measured with the suc‐AAPF‐pNA assay at 30 °C in the pH range of 5.0–12.0. The maximum activity for each protease was considered as 100% activity; SPAO (closed circles; 358 U·mg^−1^), BPN' (squares; 604 U·mg^−1^), Savinase (triangles; 705 U·mg^−1^), and subtilisin Carlsberg (open circles; 1193 U·mg^−1^). The experiments were performed in triplicates, and data are plotted as mean values ± SD.

The effects of pH on the activity of the enzymes toward the substrate suc‐AAPF‐pNA were studied in a pH range of 5.0–12.0 at 30 °C, as shown in Fig. 9. The highest activity of the protease SPAO was observed at pH 9.0–9.5, whereas at pH 6.5 and pH 12, only 7% and 60% of the maximal activity were measured, respectively. For SPAO, a stronger effect of the buffer system was observed, since a high activity rise after a buffer system change was observed. Savinase, BPN', and subtilisin Carlsberg also exhibited an optimal activity at pH 9.0. The acquired data for the reference proteases are congruent to the data found in literature [36, 57]. The pH optimum of SPAO at pH 9.0 and its working range until pH 12.0 draw attention to its great potential toward various industrial applications, similar to other alkaline proteases reported before [51, 52, 58]. Thus, SPAO and Savinase are not only highly alkaline subtilisins based on their amino acid sequence, but also differ clearly from BPN' and subtilisin Carlsberg with increased activity at pH 12.0. High‐alkaline proteases adapt to higher alkaline conditions by an altered surface charge at higher pHs, expressed by an increased pI value of the enzyme with a higher number of Arg and a decreased number of Lys [57]. This is also true for SPAO, especially when considering only the surface‐exposed residues. Six of the nine Arg residues and one of the three Lys residues of SPAO are surface‐exposed (Fig. 3, Table 2).

**Table 2:** Physicochemical parameters of the proteases.

Protease	Experimental pI	Arg	Asp	Glu	His	Lys	AB ratio^a^
SPAO	9.80	9	4	4	6	3	0.4
Savinase	9.80	8	5	5	7	5	0.5
subtilisin Carlsberg	8.00	3	9	5	5	10	0.8
BPN'	7.80^b^	2	10	5	6	11	0.8

^a^ The AB ratio [AB = (Glu + Asp)/(Lys + His + Arg)] was calculated as described in R^85^].

^b^ pI of BPN’ according to Matsubara et al. [38].

The protease SPAO showed good stability at pH 6–7 and pH 11–12 and lost more than 50% of its activity at pH 9.0, which is also the pH optimum of the protease and might be due to higher autoproteolysis, while BPN', Savinase, and subtilisin Carlsberg showed almost no remaining protease activity after 4 h (Fig. S2). Subtilisin Sendai was stable at pH 12, and it maintained 80% of its activity after 6 h at 30 °C [40]. The alkaline serine protease from *Geobacillus toebii* LBT 77 lost no activity between pH 8 and 13 after 12‐h incubation at 60 °C [46]. Likewise, ALTP from *Alkaliphilus transvaalensis* or the serine protease from *Bacillus* sp. NPST‐AK15 showed comparable stability between pH 5 and 11 after incubation for 10 min at 50 °C and 2 h at room temperature, respectively [47, 59].

### Effect of SDS and H_2_O_2_ on enzyme activity

The effect of SDS on SPAO and the reference proteases is reported in Table 1. SPAO is highly sensitive against 1% and 5% SDS (w/v) with no residual activity after 1 h at 10 °C. In contrast to that, the reference proteases showed high stability against SDS and were even more active than without. The anionic nature of SDS allows interactions between SDS and amino acid residues leading to the unfolding of the protein and loss of enzyme activity. For some subtilisins, SDS may not unfold the protein but instead helps to achieve a favorable protein conformation that stimulates activity, a behavior that was observed here for the reference proteases and has also been described in the literature. Bhatt and Singh [60] reported an activity of 275% for the alkaline serine protease from the newly isolated haloalkaliphilic *Bacillus lehensis* JO‐26 after incubation with 1% SDS for 30 min compared to the activity without SDS. Joshi and Satyanarayana [61] reported for the *Bacillus lehensis* BLAP protease an activity of 99% after incubation with 1% SDS and an increased activity 160% after incubation with 2% SDS. The protease isolated from *Geobacillus toebii* LBT 77 displayed an activity of 120% after incubation with 1% SDS for 1 h at 55 °C [46]. An alkaline protease from *Bacillus licheniformis* RP1 lost 27% activity after incubation with 0.5% SDS [62]. The alkaline protease from *Bacillus clausii* I‐52 displayed a remaining activity of 73% after incubation with 5% SDS [63]. Therefore, it is quite unusual for a high‐alkaline subtilisin to lose complete activity after incubation with SDS. Another example is the intracellular subtilase AprX‐SK37 from *Virgibacillus* sp. SK37, which showed a complete loss of activity when incubated with 0.5% SDS for 30 min at room temperature [51].

Subtilisin Carlsberg was investigated for its stability against 1% SDS for 1 h at 30 °C by Tanaka et al. [54]. In comparison with the activity increase measured in our work, they showed a complete activity loss after 60 min. Looking at the temperature stability of subtilisin Carlsberg in Fig. 8, this result might be due to autoproteolysis rather than instability to SDS. Our results are consistent with a previous report that subtilisin Carlsberg was structurally unaffected upon interaction with SDS micelles [64].

The response of SPAO to H_2_O_2_ is also shown in Table 1. After a 1‐h treatment with 1% (v/v) H_2_O_2_, subtilisin Carlsberg, Savinase, and BPN' displayed a loss of activity of 29%, 36%, and 19%, respectively. In contrast, SPAO showed a slight increase in activity of 8% at 1% (v/v) H_2_O_2_. After a 1‐h treatment with 5% (v/v) H_2_O_2_, the reference enzymes lost up to 92% activity (Savinase), while SPAO lost only 42% of its activity. SPAO is thus a highly oxidatively stable protease, especially in contrast to subtilisin Carlsberg, Savinase, and BPN'. H_2_O_2_ likely oxidizes a conserved methionine residue adjacent to the catalytic serine residue to its corresponding sulfoxide, which may lead to the inactivation of the enzyme [65]. The effect on the catalytic efficiency can be attributed to the unfavorable orientation of the sulfoxide oxygen of the oxidized methionine residue toward the oxyanion hole, thus destabilizing the tetrahedral intermediate formed with the carbonyl group of the peptide to be hydrolyzed [66, 67]. Engineering the enzyme by replacing the methionine residue 222 (BPN' numbering) has been shown to increase the resistance to oxidants [68, 69, 70, 71]. Nonaka et al. [72] showed that the digestion pattern of β‐casein cleaved by oxidized proteases differs from that cleaved by unoxidized enzymes, suggesting that oxidation of the methionine is not a fatal modification but alters the substrate specificity. This altered substrate specificity could explain the slight increase in activity observed for SPAO, which possesses also Met^216^ next to the catalytic Ser^215^.

In previous reports on the stability of alkaline proteases toward oxidants, a protease from *Bacillus clausii* I‐52 was also found to have an increase in activity by 14% and 16% at 1% and 5% H_2_O_2_, respectively [63]. The protease from the alkaliphilic *Bacillus* sp. NPST‐AK15 displayed a comparable increase of 2% at 1% H_2_O_2_ while losing 6% activity at 5% H_2_O_2_ [47]. Besides the two proteases mentioned above, the two serine proteases BM1 and BM2 derived from *Bacillus mojavensis* lost 62% and 60% activity by treatment with 5% H_2_O_2_ for 1 h at 30 °C, while a subtilase from *Thermoactinomyces vulgaris* strain CDF lost 90% activity after 1 h at 40 °C with 5% H_2_O_2_ [73, 74]. Rekik et al. [75] reported for a protease from *Bacillus safensis* RH12 (SAPRH) an activity of 160% with surprisingly high H_2_O_2_ concentrations of 15% (v/v), which was also observed for the reference proteases they used: *Bacillus pumilus* (SAPB) with 109% activity and Alcalase 2.5 L, type DX with 150% activity. In contrast, the subtilase KP‐43 from the group of oxidatively stable proteases (OSP) lost its ability to hydrolyze after only 30‐min incubation with 3% H_2_O_2_ [72]. However, comparisons with literature data are often difficult because the buffers, temperatures, pH values, H_2_O_2_ concentration, and substrate for the activity studies are different.

Proteases can be classified by their sensitivity to various inhibitors [76]. Incubation of SPAO and the three reference proteases with 1 mm PMSF led to a complete inhibition of all proteases (Table 1).

### Effects of NaCl, chelating agents (EDTA), and Ca^2+^ on enzyme activity and stability

Members of the subtilase superfamily are calcium‐dependent, and the binding of Ca^2+^ is essential for enzyme stability and/or activity [77]. They usually contain two Ca^2+^ binding sites, the first being a strong binding site and the second a weak binding site [4].

The occupancy of these sites depends on the calcium ion concentration in the solution. The first site is always fully occupied even without the addition of CaCl_2_ in the solution, while the second site is occupied by an Na^+^ or K^+^ ion at low CaCl_2_ concentrations [77]. The *in silico* identification of the Ca^2+^ binding sites in SPAO revealed two Ca^2+^ binding sites as known for the three reference proteases [36]. Therefore, the effect of Ca^2+^ on the activity of the proteases was studied. All proteases, except subtilisin Carlsberg, lost more activity by incubating with EDTA before the supplementation with CaCl_2_ than without EDTA (data not shown). However, for BPN', there is almost no difference in activity between incubation with or without EDTA. For SPAO and Savinase, it was not possible to fully recover activity after the addition of CaCl_2_. In general, Ca^2+^ seems to be firmly bound, as the proteases were dissolved in a Ca^2+^‐free buffer and it was not necessary to add Ca^2+^ for their activity. At a concentration of 10 mm CaCl_2_ and above, the activity of all proteases decreased. Similar observations of the inhibitory effect of Ca^2+^ were also made for the alkaline protease from the haloalkaliphilic bacterium sp. AH‐6 [78]. The failure to recover activity could be due to a general loss of activity due to higher protein instability during incubation with EDTA. The binding of Ca^2+^ has a stabilizing effect on the protease by reducing molecular flexibility, which reduces thermal denaturation and autolysis [77]. No effect on the activity of a serine protease from *B. clausii* GMBAE 42 after incubation with EDTA and a slight inhibition by Ca^2+^ was also found by Kazan et al. [49]. Others could show a strong activity decrease after the incubation with EDTA [58, 79, 80]. However, most studies found a comparable decline in activity as seen in this work [46, 81, 82]. The findings suggest a Ca^2+^ dependency for SPAO and a good stability.

**Fig. 10:**
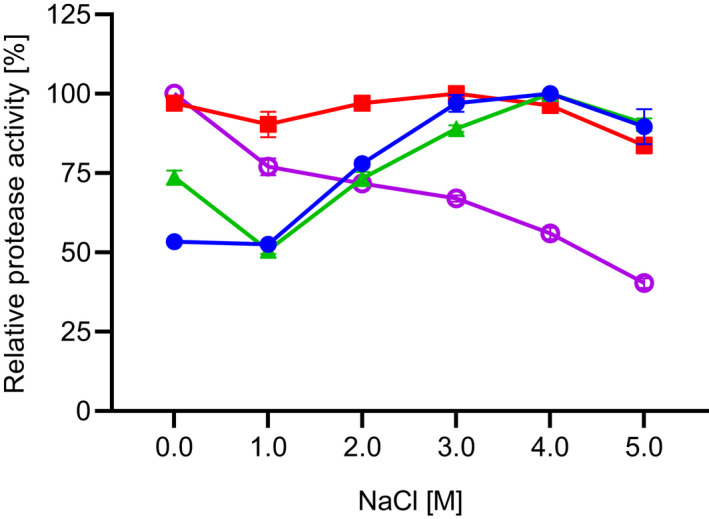
Effect of NaCl on the activity of the purified SPAO, BPN', Savinase, and subtilisin Carlsberg. The activity was measured in standard buffer (pH 8.6) for suc‐AAPF‐pNA assay at 30 °C with different NaCl concentrations of 0–5 m. The maximum activity for each protease was considered as 100% activity; SPAO (closed circles; 1205 U·mg^−1^), BPN' (squares; 560 U·mg^−1^), Savinase (triangles; 757 U·mg^−1^), and subtilisin Carlsberg (open circles; 846 U·mg^−1^). The experiment was performed in triplicates, and data are given as mean values ± SD.

**Fig. 11:**
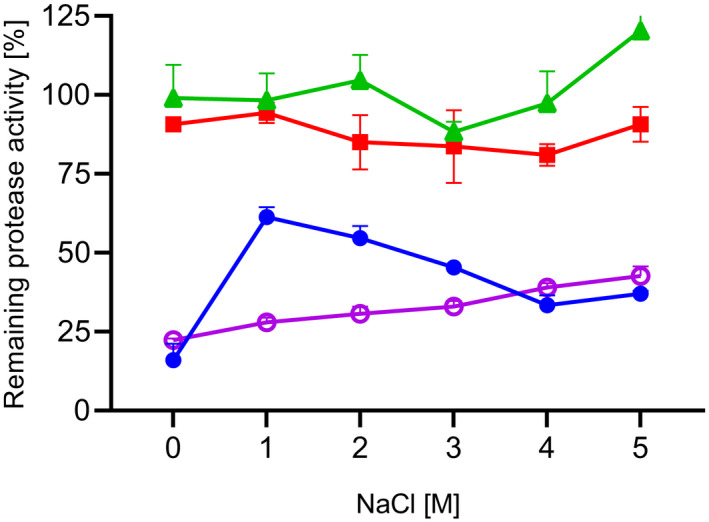
Stability against NaCl of purified SPAO, BPN', Savinase, and subtilisin Carlsberg. Stability was tested in 10 mm HEPES/NaOH buffer, pH 8.0, with different NaCl concentrations (0–5 m) after 2 h at 20 °C. The activity was measured with the suc‐AAPF‐pNA assay in standard buffer at pH 8.6. Activity at 0 h for each NaCl concentration was considered as 100% activity; SPAO (circles), BPN' (squares), Savinase (triangles), and subtilisin Carlsberg (open circles). The experiment was performed in triplicates, and data are given as mean values ± SD.

The effect of NaCl on the activity of the proteases was evaluated in the activity assay using the substrate suc‐AAPF‐pNA and different concentrations of NaCl (0–5 m) in standard reaction buffer (pH 8.6). The proteolytic activity is shown in Fig. 10. SPAO showed increased activity with rising NaCl concentrations up to the maximum at 4 m NaCl, followed by a 10% decrease at 5 m NaCl. Savinase showed the highest activity at 3 and 4 m NaCl. BPN' displayed high activity from 0 to 4 m NaCl, which was reduced to 77% at 5 m NaCl. The stability of SPAO and the three reference proteases was examined by incubation with NaCl concentration between 0 and 5 m NaCl in 10 mm HEPES/NaOH, pH 8.0, at 20 °C for 2 h (Fig. 11). The results show that Savinase and BPN' were stable with and without NaCl. SPAO and subtilisin Carlsberg were stabilized by NaCl. However, SPAO showed stability above 50% only with 1 m NaCl while losing stability with increasing NaCl concentrations.

In contrast to halophilic enzymes, halotolerant enzymes are active over a broad range of NaCl concentrations and retain their activity even in the absence of NaCl [83]. Unlike the mechanism of halophilic enzymes, the mechanism of halotolerance is not yet fully elucidated [79]. Halophilic proteins have a predominance of negatively charged residues on the solvent‐exposed surface, which attract water molecules and thereby keep the enzymes hydrated [84]. Therefore, a forecast to salt adaptation could be made based on the ratio of the acidic amino acids Glu and Asp to the basic amino acids Lys, His, and Arg (AB ratio) and a more acidic isoelectric point [84, 85]. While SPAO and Savinase share a higher pI, the AB ratio is lower than for BPN' and subtilisin Carlsberg, which indicates a poor adaptation to salt (Table 2). However, the high Arg content of SPAO and Savinase, which favors adaptation to highly alkaline conditions, results in a predominantly positive charge on the protein surface in SPAO (Fig. 4), and in general, a high proportion of negative or positive charges on the surface of the enzyme improves salt adaptation [86]. BPN' has five of the seven amino acid positions identified by Takenaka et al. [86] as favorable for salt adaptation and therefore, unlike subtilisin Carlsberg, shows good adaptation to high salt concentrations.

### Proteolytic activity on synthetic peptides

In general, data for proteinase specificity under comparable experimental conditions are limited in the literature. Usually, kinetic data for the hydrolysis of synthetic substrates are collected. To analyze the substrate specificity of SPAO and the three reference proteases, 10 synthetic three or four amino acid peptide‐4‐nitroanilide substrates were chosen, which are typical subtilisin substrates [87]. However, suc‐AAA‐pNA is a typical elastase substrate [88]. As shown in Table 3, SPAO showed the highest specific activity for suc‐FAAF‐pNA and a very low specificity for suc‐TVAA‐pNA and suc‐YVAD‐pNA under the selected conditions, which is in good agreement with the results obtained for the typical subtilisins Savinase, subtilisin Carlsberg, and BPN'.

**Table 3:** Substrate specificities of the proteases against 10 synthetic substrates (suc‐XXXX‐pNA). Kinetic experiments were carried out in 0.1 m Tris/HCl buffer, pH 8.6, and 0.1% (w/v) Brij®35 over 5 min at 30 °C with 17 mm of a substrate. The experiment was performed in triplicates, and the standard deviation was < 5%. The enzyme activity against AAPF refers to 100% relative activity.

Protease	Relative activity [%]									
	FAAF	AAA	AAVA	ALPF	AGPF	AAPF	TVAA	YVAD	AGPP	AAPL
SPAO	753	12	21	127	107	100 (42 U·mg^−1^)	3	3	216	34
Subtilisin Carlsberg	57	0	2	60	90	100 (570 U·mg^−1^)	1	1	147	104
Savinase	605	8	22	117	96	100 (180 U·mg^−1^)	5	5	144	12
BPN'	96	0	6	106	96	100 (181 U·mg^−1^)	0	0	61	67

In general, subtilisins show a broad substrate specificity and often display a preference for large hydrophobic groups at the P1 position, the first position N‐terminal to the cleavage site (nomenclature of Schechter and Berger [89]), here to the 4‐nitroanilide (P1'), which can also be observed in this experiment [90]. However, the protease SPAO and Savinase are able to hydrolyze the substrate if alanine is at the P1 and P2 position, but with higher efficiency, if one of the positions is alanine and an amino acid with a larger hydrophobic group. In literature, data for the hydrolysis of synthetic substrates are collected under different conditions, which makes the results difficult for comparison and interpretation. Kazan et al. [49] reported an alkaline serine protease from *B. clausii* GMBAE 42 with high specificity for suc‐AAPF‐pNA. Georgieva et al. [87] reported for Savinase also the highest specificity for suc‐FAAF‐pNA by comparing it with Esperase with a similar preference. Proteinase K showed the highest specificity for suc‐AGPF‐pNA [91]. For the elastase‐specific substrate suc‐AAA‐pNA, SPAO showed low activity in contrast to the alkaline elastase YaB from *Bacillus* strain YaB [92]. In the context of this experiment and also in line with the high sequence similarity to Savinase (82.16%), SPAO can be considered a typical subtilisin.

## Conclusion

In this study, we recombinantly expressed SPAO, a novel high‐alkaline subtilisin isolated from *Alkalihalobacillus okhensis* Kh10‐101^T^. SPAO was effectively produced and secreted by *B. subtilis* DB104. After purification, biochemical characterization revealed a highly oxidatively stable protease with increased activity upon incubation with 1% H_2_O_2_ and residual activity of 58% with 5% H_2_O_2_. The high Arg content and the predominantly positive charge on the protein surface allow SPAO to be highly active at pH 12.0 and at NaCl concentrations of up to 5 m. The optimal temperature and pH were 55 °C and pH 9.0–10.5, respectively. With its biochemical properties, SPAO shows potential for industrial applications to be evaluated in the future.

## Conflict of interest

The authors declare no conflict of interest.

## Author contributions

FF, JB, and PS conceived and designed the experiments. FF collected and analyzed the data. DF and JR carried out the cloning and pre‐experiments. FF wrote the original draft. FF, JB, MB, and PS revised the manuscript. All authors contributed to the final manuscript.

## Supporting information


**Table S1.** Preculture medium.
**Table S2.** Fermentation medium.
**Table S3.** Trace element solution.
**Fig. S1.** MALDI‐TOF mass spectra of SPAO.
**Fig. S2.** Effect of pH on the stability of purified SPAO, BPN', Savinase, and subtilisin Carlsberg.

## Data Availability

The data that support the findings of this study are available from the corresponding author [siegert@fh-aachen.de] upon reasonable request.
