# Supplementary material for: Biochemical characterization of a novel oxidatively stable, halotolerant, and high‐alkaline subtilisin from Alkalihalobacillus okhensis Kh10‐101T
Source: FEBS Open Bio. 2022 Jul 6;12(10):1729–46. doi: 10.1002/2211-5463.13457 (PMC9527586; doi:10.1002/2211-5463.13457)
Supplement: Supplementary file 1 — Table S1. Preculture medium. Table S2. Fermentation medium. Table S3. Trace element solution. Fig. S1. MALDI‐TOF mass spectra of SPAO. Fig. S2. Effect of pH on the stability of purified SPAO, BPN', Savinase, and subtilisin Carlsberg. [file FEB4-12-1729-s001.docx]

# Supplementary

Table S1. Preculture medium

| **Component** | **Concentration** |
| --- | --- |
| MgSO_4_ | 0.7 g·L^-1^ |
| KH_2_PO_4_ | 7.7 g·L^-1^ |
| (NH_4_)_2_SO_4_ | 2.8 g·L^-1^ |
| FeSO_4_ x 7H_2_O | 0.09 g·L^-1^ |
| MnSO_4_ x H_2_O | 0.09 g·L^-1^ |
| Peptone from soy | 40 g·L^-1^ |
| Yeast extract | 5 g·L^-1^ |
| Tetracycline | 20 µg·mL^-1^ |
| CaCl_2_ | 1 g·L^-1^ |
| Glucose | 20 g·L^-1^ |
| Trace elements | see Table S 3 |

Table S2. Fermentation medium

| **Component** | **Concentration** |
| --- | --- |
| MgSO_4_ | 0.7 g·L^-1^ |
| KH_2_PO_4_ | 7.7 g·L^-1^ |
| (NH_4_)_2_SO_4_ | 2.8 g·L^-1^ |
| FeSO_4_ x 7H_2_O | 0.09 g·L^-1^ |
| MnSO_4_ x H_2_O | 0.09 g·L^-1^ |
| Peptone from soy | 40 g·L^-1^ |
| Yeast extract | 5 g·L^-1^ |
| Tetracycline | 20 µg·mL^-1^ |
| CaCl_2_ | 1 g·L^-1^ |
| Glucose | 8 g·L^-1^ start glucose + glucose feed |
| Trace elements | see Table S 3 |
| Polypropylene glycol 2000 (PPG) | 2 mL + PPG feed |

Table S3. Trace element solution

| **Chemical** | **Concentration** |
| --- | --- |
| Citric acid_*_H_2_O | 40 g·L^-1^ |
| MnSO_4*_H_2_O | 4 g·L^-1^ |
| ZnSO_4*_7H_2_O | 5 g·L^-1^ |
| CuSO_4*_5H_2_O | 4 g·L^-1^ |
| FeSO_4*_7H_2_O | 10.67 g·L^-1^ |
| NiSO_4*_6H_2_O | 0.25 g·L^-1^ |
| CoCl_2*_6H_2_O | 0.324 g·L^-1^ |
| H_3_BO_3_ | 0.06 g·L^-1^ |
| Na_2_MoO_4*_2H_2_O | 0.655 g·L^-1^ |


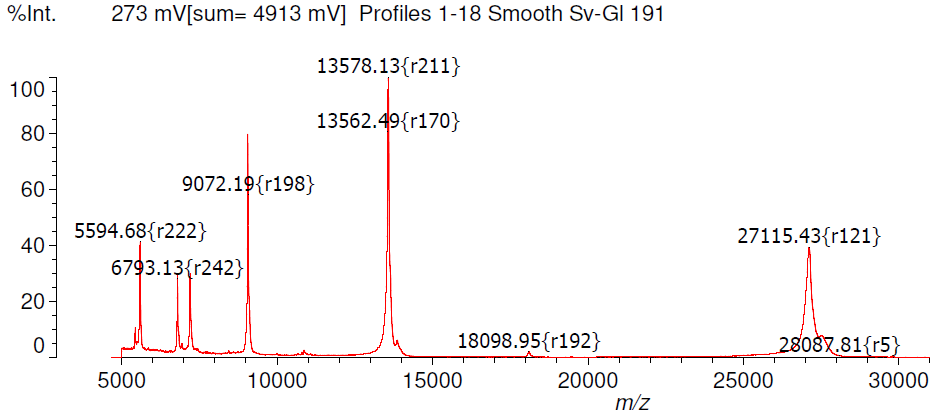


Fig. S1. MALDI-TOF mass spectra of SPAO. The labels on the peaks indicate the measured average molecular mass. The peaks correspond from right to left M/z up to M/5z.


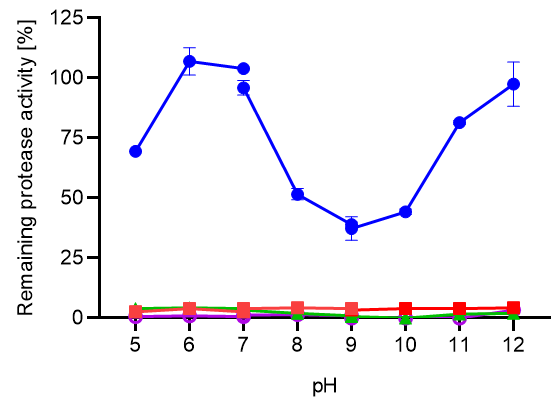


Fig. S2. The effect of pH on the stability of purified SPAO, BPN’, Savinase, and subtilisin Carlsberg. The activity was measured with the suc-AAPF-pNA assay in standard buffer at pH 8.6 after incubation for 4 h at 4°C in Tris-maleate buffer (pH 5 – 7), in Tris-HCl (pH 7 – 9), and in glycine-NaOH (pH 9 – 12). The activity at 0 h was considered as 100 % activity; highest residual activities: SPAO (closed circles; 288 U/mg), BPN’ (squares; 17 U/mg), Savinase (triangles; 15 U/mg) and subtilisin Carlsberg (open circles; 23 U/mg). The experiments were performed in triplicates and data are plotted as mean values ± SD.
